# A case of primary cardiac sarcoma with an acute presentation: The role of multimodality imaging

**DOI:** 10.1002/ccr3.4219

**Published:** 2021-06-17

**Authors:** Luca Restivo, Antonio De Luca, Bruno Pinamonti, Giulia Grilli, Rossana Bussani, Franco Cominotto, Carmelo Crisafulli, Franca Dore, Gianfranco Sinagra, Aniello Pappalardo

**Affiliations:** ^1^ Division of Cardiology Cardiothoracovascular Department Azienda Sanitaria Universitaria Giuliano‐Isontina and University of Trieste Trieste Italy; ^2^ Pathology Department Azienda Sanitaria Universitaria Giuliano‐Isontina and University of Trieste Trieste Italy; ^3^ Emergency Medicine Department Azienda Sanitaria Universitaria Giuliano‐Isontina Trieste Italy; ^4^ Nuclear Medicine Department Azienda Sanitaria Universitaria Giuliano‐Isontina Trieste Italy; ^5^ Division of Cardiac Surgery Cardiovascular Department Azienda Sanitaria Universitaria Giuliano‐Isontina Trieste Italy

**Keywords:** cardiac masses, cardiac sarcoma, cardiac tumors

## Abstract

The case highlights the value of contrast echocardiography in raising clinical suspicion of malignancy, allowing a diagnostic work‐up and the treatment of the primitive heart tumors.

## Graphical abstract

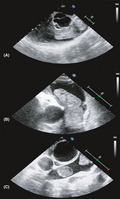

## 1 INTRODUCTION

Primary cardiac sarcomas are the most commonly encountered primary cardiac malignancies with a marked tendency of recurrence. In the complex and heterogeneous field of cardiac masses, a proper differential diagnosis based on multimodality imaging approach is extremely useful in order to plan the most appropriate treatment.

Primary cardiac tumors are rare and can cause a broad spectrum of unexpected symptoms and clinical manifestations. Primary cardiac sarcomas constitute approximately 1% of all soft tissue sarcomas and are the most common malignant primary cardiac tumor.^1^, ^2^ They occur most commonly in the left atrium, but can develop in any cardiac chamber.^3^

We report the case of 69‐year‐old woman presenting at the emergency department (ED) for worsening dyspnea, syncope, and acute right heart failure due to a mass in the right ventricular outflow tract (RVOT) causing pulmonary obstruction.

## 2 CASE PRESENTATION

A 69‐year‐old obese woman with no previous cardiac history was admitted to Emergency Department for syncope and recent onset of worsening dyspnea. Physical examination showed signs of peripheral congestion and revealed a new systolic heart murmur. The patient was normotensive and the ECG was nonspecific. Blood test showed a slight increase of cardiac troponin I and D‐dimer.

**FIGURE 1:**
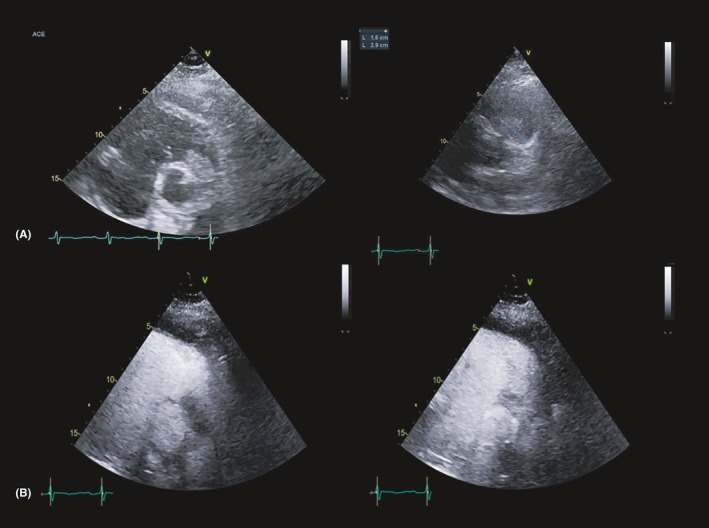
A, transthoracic echocardiography, parasternal short‐axis view. Hypoechoic mass at the level of right ventricular outflow tract is evident. B, contrast‐enhanced transthoracic echocardiography. Left: early acquisition after contrast administration, hypoechoic aspect of the mass. Right: late acquisition with significant enhancement of the mass

The patient underwent transthoracic echocardiography (TTE) that showed right ventricular dilatation and dysfunction with McConnell's sign and a mobile mass was observed in the RVOT (Figure 1A), which engaged the origin of the pulmonary artery. A subvalvular pulmonary stenosis with moderate to severe systolic RVOT gradient (peak/mean gradient: 54/22 mm Hg) and high‐velocity jet of tricuspid regurgitation (peak vel. 4.4 m/s) were also documented. Paradoxical septal motion and left ventricular D‐shape were also evident.

In the suspicion of acute pulmonary embolism, a chest CT angiography was performed, which confirmed the finding and documented another filling defect of the right branch of the pulmonary artery. Thus, heparin therapy was started. However, despite effective anticoagulant therapy, the mass persisted.

**FIGURE 2:**
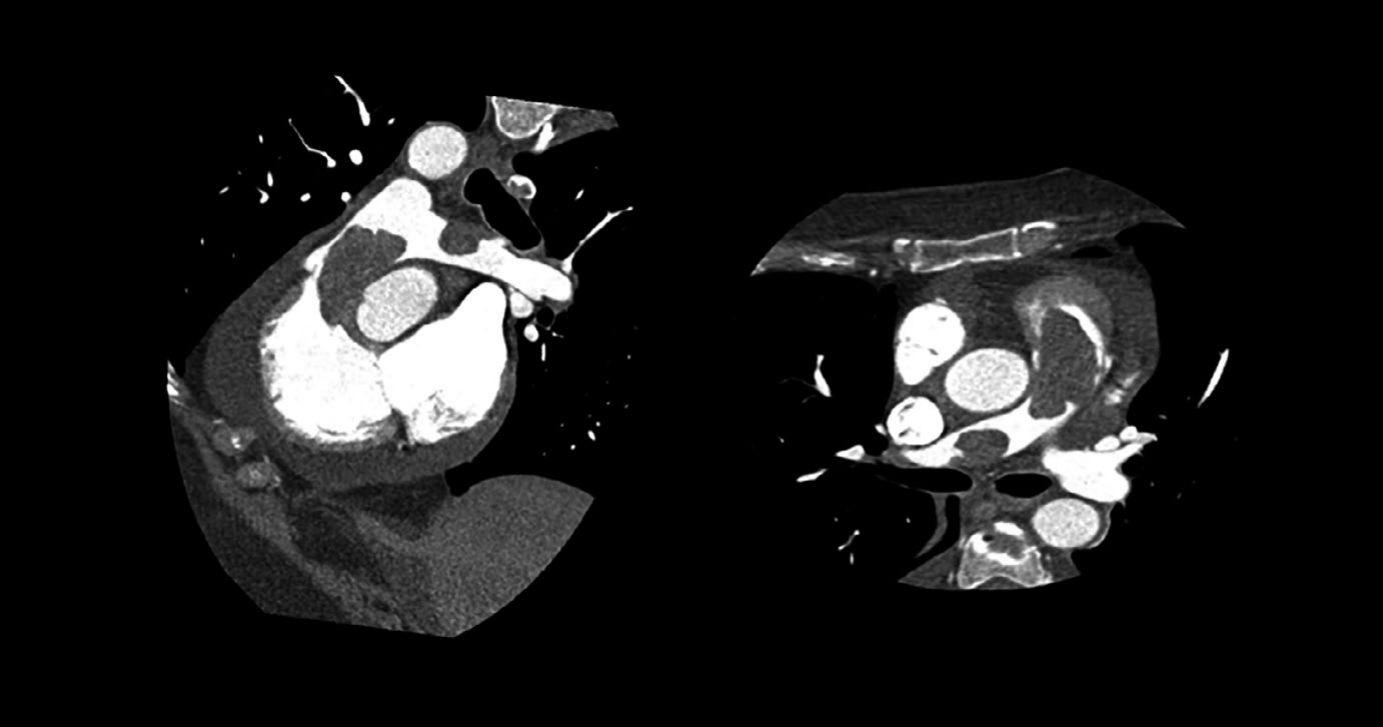
Cardiac computed tomography reconstructions showing the two masses involving the right ventricular outflow tract and the right branch of pulmonary artery

**FIGURE 3:**
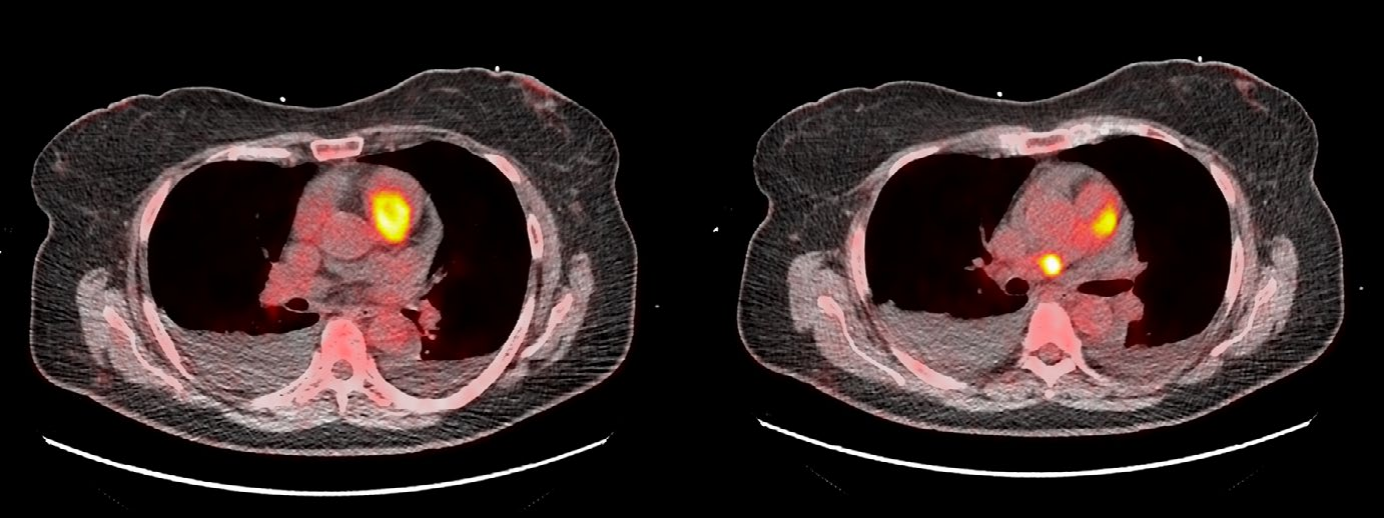
18‐fluorodeoxyglucose positron emission tomography/computed tomography (18‐FDG PET/CT). Hypercaptation of the two masses, consistent with elevated metabolic activity

To improve diagnostic characterization of the mass, contrast echocardiography was performed, showing a complete opacification of cardiac chambers with late inhomogeneous enhancement of the mass, raising the hypothesis of cardiac tumor (Figure 1B). Since cardiac magnetic resonance was contraindicated due to the presence of cochlear implant, a cardiac‐CT was performed in order to better characterize the mass. The CT scan showed a hypodense formation with origin from the wall of RVOT and confirmed the presence of a second mass in the right branch of pulmonary artery (Figure 2). Both the formations showed significant contrast enhancement, highly suggestive of neoplastic nature. 18‐fluorodeoxyglucose positron emission tomography/computed tomography (18‐FDG PET/CT) confirmed the high metabolic activity of the masses and excluded other extracardiac pathologic captations (Figure 3).

**FIGURE 4:**
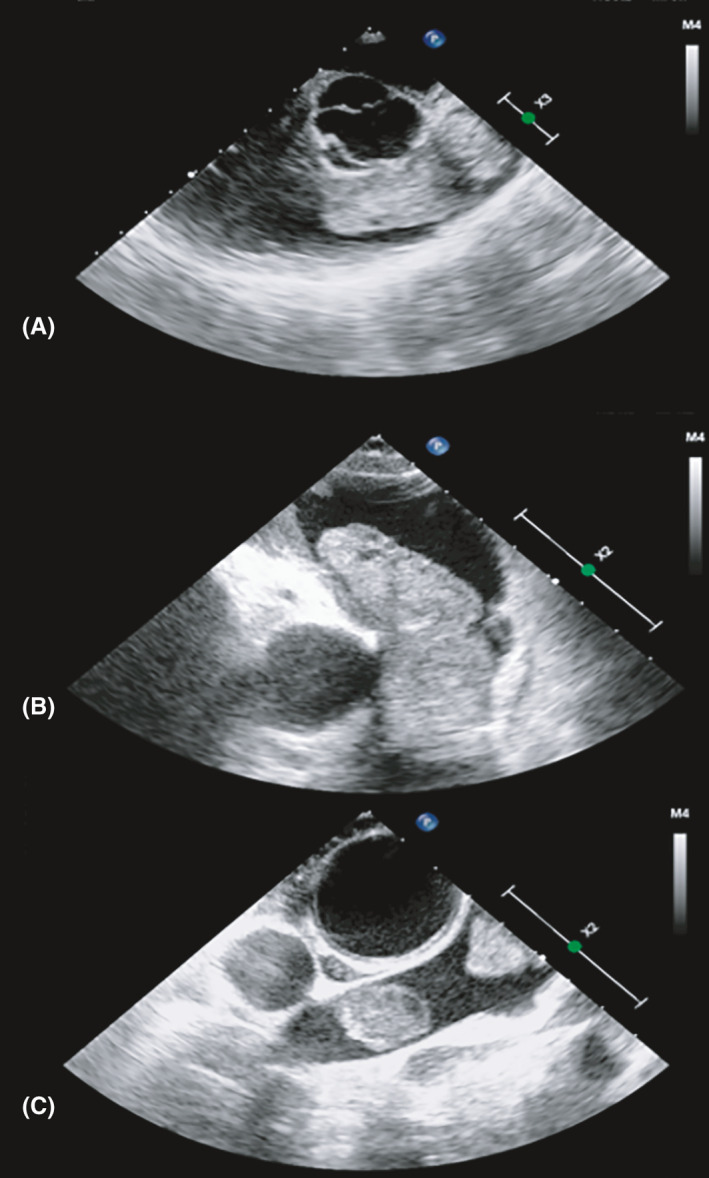
Intraoperative transoesophageal echocardiography. A and B, coarse and inhomogeneous mass of the right ventricular outflow tract. C, mass involving the right branch of the pulmonary artery

**FIGURE 5:**
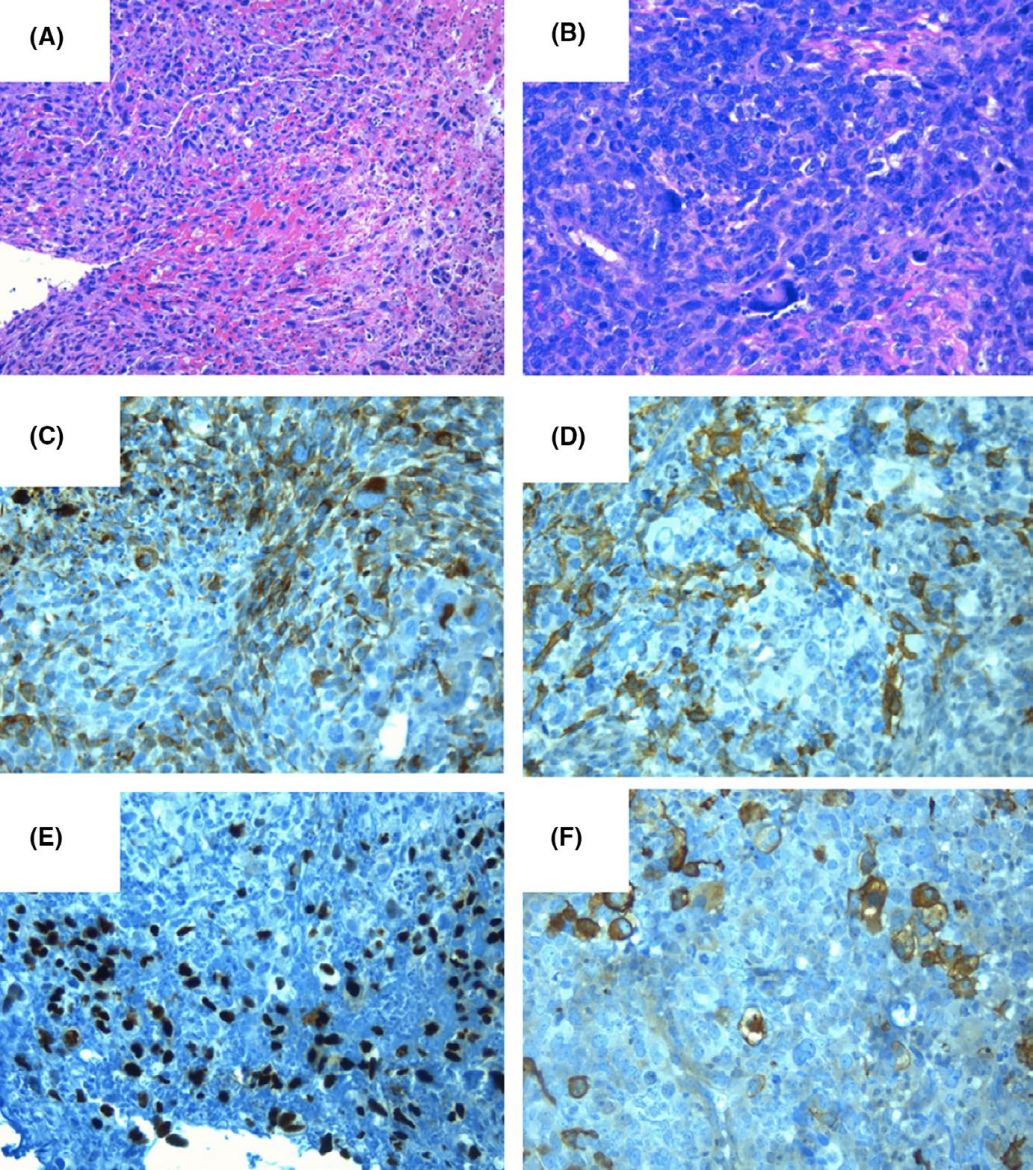
Histological samples of the lesion, which consists of highly pleomorphic, large tumor cells with marked atypia with a solid pattern and hemorrhagic areas (A, Hematoxylin and Eosin ×10. B, Hematoxylin and Eosin ×20). Some cells showed no reactivity to any immunohistochemical marker, while others were positive for muscle markers showing focal skeletal muscle differentiation (C, Desmin ×20. D, SMA ×20. E, Myo D1 ×20. F, HHF35 ×20)

After Heart Team discussion, surgical mass debulking was planned. Intraoperative transoesophageal echocardiography showed a first vacuolated and inhomogeneous mass (5.2 × 2.7 cm) with irregular surface, obstructing the RVOT and pulmonary trunk​ (Video S1), and a second mass (2.2 × 1.4 cm) in the right branch of pulmonary artery (Figure 4). The exeresis was macroscopically radical, despite the dimensions, friability, and local invasivity of the masses. Since the pulmonary valve and the right pulmonary artery were infiltrated by the tumor, both of them were resected and replaced with a bioprosthetic valve and a vascular prosthesis, respectively. The histologic specimen examination revealed an undifferentiated primary mesenchymal neoplasm consistent with pleomorphic sarcoma with an elevated mitotic index (Ki‐67 > 80%) with coexisting immunophenotypic traits guiding for dedifferentiated leiomyosarcoma (Figure 5). At postoperative echocardiography, there was no evidence of intraventricular mass. Subsequently, the patient started an adjuvant chemotherapy with epidoxorubicin and had close oncological follow‐up.

## 3 DISCUSSION

Primary cardiac tumors are rare, with an overall incidence of 0.056%‐0.02% of autopsy series.^4^ Cardiac myxomas represent the most common benign cardiac tumor.^5^ Other tumors include rhabdomyomas, fibromas, papillary fibroelastomas, hemangiomas, lipomas, hamartomas, teratomas, mesotheliomas, and paragangliomas. Moreover, various types of sarcomas can be found, such as angiosarcoma (which most often originates in the right atrium), myxosarcoma, liposarcoma, fibrosarcoma, leiomyosarcoma, osteosarcoma, synovial sarcoma, rhabdomyosarcoma, undifferentiated sarcoma, reticulum cell sarcoma, neurofibrosarcoma, and malignant fibrous histiocytoma.^4^, ^5^

Undifferentiated pleomorphic sarcomas are the most commonly encountered primary cardiac malignancy, accounting for approximately 10% of all primary cardiac tumors.^6^ The mean age at presentation is 45 years, with no sexual predilection. They occur most commonly in the left atrium, but can develop in any cardiac chamber.^3^

Cardiac masses can be effectively identified and characterized by multimodality imaging. TTE remains the first diagnostic approach and permits to evaluate site of origin, size, mobility, and hemodynamic impact of the mass.^6^ Contrast‐TTE is useful to assess the perfusion of the mass in order to differentiate vascular tumor from thrombi. Malignant tumors are frequently highly vascularized and show significant contrast enhancement, whereas, myxomas demonstrate mild enhancement by contrast on visual inspection and quantitatively less perfusion than the surrounding myocardium. Thrombi, being avascular, show complete absence of contrast opacification.^7^ CT can provide additional useful information, such as better definition of site of origin, anatomical relationships, extension to surrounding structures, and evaluation of extracardiac localizations.^8^ CMR is the best available noninvasive diagnostic tool to provide information about morphology, dimensions, location, extension, perfusion, and tissue characterization of the mass, orienting toward the histopathological diagnosis.^8^ 18F‐FDG offers an accurate evaluation of the metabolic activity of tumors. The extent of FDG uptake by tumors improves differentiation between benign and malignant tumors. 18F‐FDG could also be useful for staging malignancies and in the evaluation of early responses to cancer therapy.^9^

**FIGURE 6:**
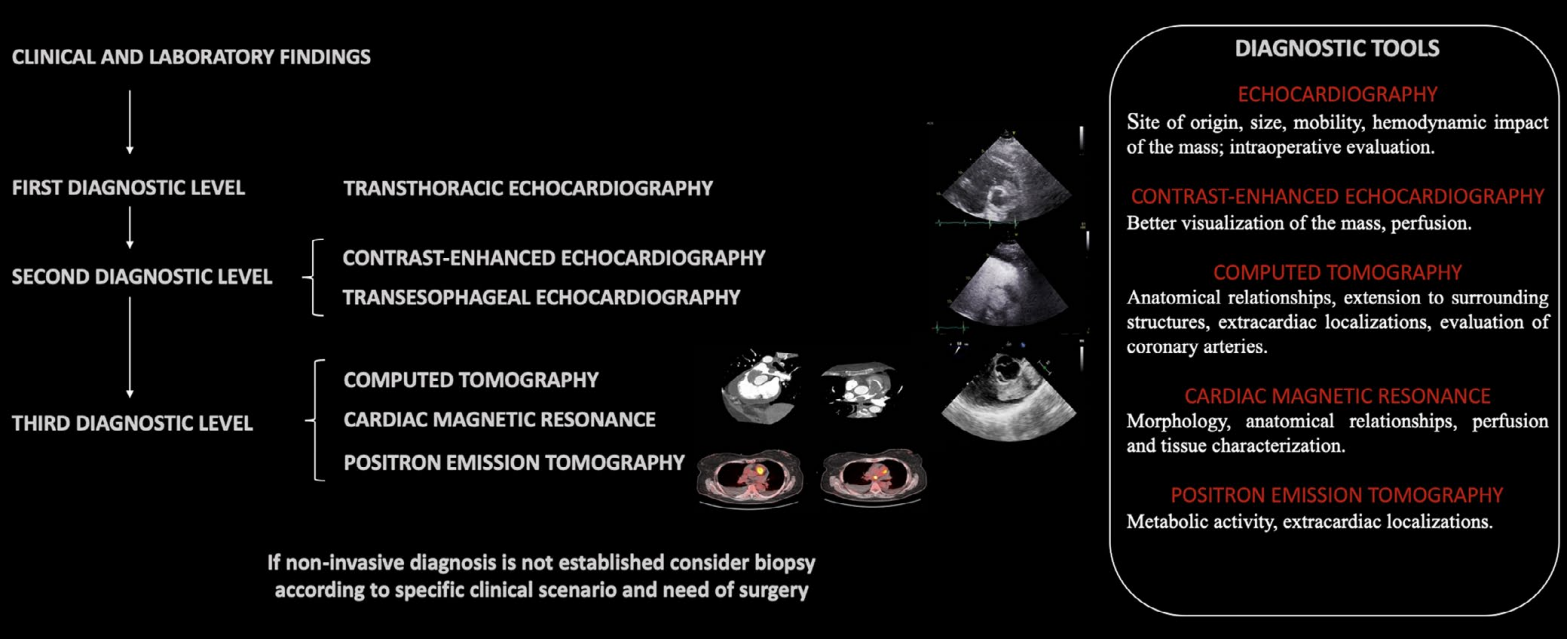
Proposed diagnostic work‐up for diagnosis through multimodal imaging

In the complex field of cardiac masses, a multimodality imaging approach is crucial to reach an early and true diagnosis (Figure 6). Nevertheless, histopathology remains the diagnostic gold standard in any resected cardiac mass, allowing to establish the benign or malignant nature and the precise histotype.^6^

Optimal treatment approach for these neoplasms is still unclear. Surgery when feasible is the best therapeutic strategy.^10^, ^11^ A complete surgical resection of the malignancy was found to be significantly associated with longer survival (17 vs 6 months when complete resection was not possible).^12^ The benefit of adjuvant chemotherapy and/or radiation is unknown, but is a treatment option, especially for patients with incomplete resections. Furthermore, the role of radiotherapy (RT) is limited, considering that high‐dose radiation protocols used to treat sarcomas in other locations are poorly tolerated by the heart.^12^, ^13^ It has to be noted that, despite radical exeresis of the mass, local recurrence and metastasis within 1 year are frequent and the median progression‐free survival is 5.9 months,^13^, ^14^ ranging from 6 to 18 months.^12^ To date, since a clear evidence of adjuvant treatments effectiveness is lacking, surgery must be considered the pivotal therapy for a successful management.^15^ Future efforts should be directed to the improvement of surgical techniques to permit safe radical excision and to the development of effective adjuvant therapy.

## 4 CONCLUSION

Neoplastic cardiac masses require an integrated diagnostic‐therapeutic work‐up to guarantee a tailored therapy. In the complex field of cardiac masses, a prompt and accurate diagnosis are crucial but challenging and often the definite diagnosis may requires histologic examination. Cardiac sarcomas are associated with poor prognosis, especially in case of metastatic disease. Optimal treatment for these neoplasms is still unclear and surgery continues to be the best treatment option.

## CONFLICT OF INTEREST

None declared.

## AUTHOR CONTRIBUTIONS

All authors involved in drafting the manuscript (LR, ADL, BP, GG, RB, FC, CC, FD, GS and AP) or revising it critically (especially thanks to GS and RB) for important intellectual content.

## CONSENT STATEMENT

Published with written consent of the patient.

## ETHICAL APPROVAL

The study was conducted according to the declaration of Helsinki.

## DISCLOSURES

The authors have nothing to disclose.

## Supporting information

Supplementary Material

## Data Availability

Data sharing not applicable to this article as no datasets were generated or analyzed during the current study.
